# Reduced Information Transmission of Medial Prefrontal Cortex to Basolateral Amygdala Inhibits Exploratory Behavior in Depressed Rats

**DOI:** 10.3389/fnins.2020.608587

**Published:** 2020-12-03

**Authors:** Chengxi Qi, Zihe Wang, Wenwen Bai, Tiaotiao Liu, Xuyuan Zheng

**Affiliations:** School of Biomedical Engineering and Technology, Tianjin Medical University, Tianjin, China

**Keywords:** depression, medial prefrontal cortex, basolateral amygdala, local field potentials, information flow

## Abstract

Depression is a mental and neurological disease that reduces the desire for exploration. Dysregulation of the information transmission between medial prefrontal cortex (mPFC) and basolateral amygdala (BLA) is associated with depression. However, which direction of information transmission (mPFC-BLA or BLA-mPFC) related to the decline of exploratory interests in depression is unclear. Therefore, it is important to determine what specific changes occur in mPFC and BLA information transmission in depressed rats during exploratory behavior. In the present study, local field potentials (LFPs) were recorded via multi-electrodes implanted in the mPFC and BLA for the control and depression groups of rats when they were exploring in an open field. The theta band was determined to be the characteristic band of exploratory behavior. The direct transfer function (DTF) was used to calculate the mPFC and BLA bidirectional information flow (IF) to measure information transmission. Compared with the control group, the theta IF of mPFC-BLA in the depression group was significantly reduced, and there was no significant difference in theta IF of BLA-mPFC between the two groups. Our results indicated that the reduction of mPFC-BLA information transmission can inhibit the exploratory behavior of depressed rats.

## Introduction

The lifetime prevalence (∼ 17%) and financial burden ($ 100 billion per year) associated with depression make it one of the most common and debilitating mental and neurological diseases worldwide (Ashtari et al., 1999; Pezawas et al., 2005). Depression is characterized by several psychophysiological symptoms, such as feelings of hopelessness, low energy and decreased interest in exploration (Nestler et al., 2002).

The medial prefrontal cortex (mPFC) is involved in the expression and regulation of depression-like behaviors (Nikulina et al., 2004). Neurons in the mPFC are sensitive to stress, and as a central hub, they can shape activities in a distributed network of output structures, including regulating behaviors and autonomous responses to stress (Liston et al., 2006; Czeh et al., 2007; Dias-Ferreira et al., 2009). Rodent exposure to stress can lead to mPFC dendritic spines loss and decreased activity (Covington et al., 2010). The amygdala is commonly involved in regulating negative memory and stimuli (Hendler et al., 2003), and the basolateral amygdala (BLA) is thought to be related to fear, anxiety and depression (Tye et al., 2011; Felix-Ortiz et al., 2013). Repeated stress will increase the firing rate of BLA neurons in rats, and increased membrane excitability can be observed between the main neurons of the BLA (Padival et al., 2015). The projection from the mPFC to the BLA forms a circuit that involves many cognitive and emotional processes. Although the mPFC sends excitability prediction signals to the BLA, *in vivo* electrical stimulation of the mPFC in rats have been shown to cause suppression of BLA output (Shansky et al., 2009). Under normal conditions, the mPFC exerts inhibitory control on the activity of the BLA, limiting its output and thereby preventing negative expression of emotions (Quirk et al., 2003; Motzkin et al., 2015). Electrophysiological records in the brains of depressed mice indicate that the glutamate projection synapses between the mPFC and BLA are reduced (McGarry and Carter, 2017). These findings suggest that the directional interaction between mPFC and BLA may regulate BLA activity, and the appearance of depression-like behaviors may be related to the reduction in directional activity between mPFC and BLA. Here, we employ a novel informatics approach to test this hypothesis.

Exploration is one of the basic behavioral responses of animals in the process of adapting to the environment. It can be used to obtain information about the surrounding environment and can optimize the escaping from predators (Alvarez and Alvarez, 2008; Magalhaes et al., 2008). The mPFC and BLA also play crucial roles in the exploration behavior of animals. Research indicates that the theta power of the mPFC and BLA in the safe state is higher than that in the fear state (Adhikari et al., 2010). Evidence shows that the mPFC and BLA use theta oscillations to communicate during and after fear regulation (Likhtik et al., 2014). There is evidence that the mPFC and BLA are more synchronized in the theta band in animals that distinguish between aversion and safety (Adhikari et al., 2010). Exploratory activity declines in rodents exposed to chronic stress, and theta power and synchronization of the mPFC and BLA decrease (Jacinto et al., 2013). Given the essential role of the mPFC and BLA in depression and exploratory behavior, it is important to identify what specific changes occur in mPFC and BLA information transmission in depressed rats during exploratory behavior.

To address this issue, we recorded the local field potentials (LFPs) from the mPFC and BLA in freely moving rats in the open field test (OFT). LFP has a high spatiotemporal resolution and is a powerful tool for analyzing networks in the brain structure (Marceglia et al., 2007). The OFT is commonly used to detect rodent exploratory behavior and motor activity; it can quantitatively and qualitatively detect animal activity and is widely used to detect animal models of depression (Belovicova et al., 2017). The bidirectional information flow (IF) between the mPFC and BLA network was calculated during the exploratory behavior. Furthermore, the difference in information transmission in the mPFC and BLA network between the two groups of rats was quantitatively compared.

## Materials and Methods

All experimental procedures described here were conducted in accordance with the Guide for the Care and Use of Laboratory Animals and approved by the Tianjin Medical University Animal Care and Use Committee.

### Subjects and Chronic Unpredictable Mild Stress (CUMS) Procedure

The experimental animals were 12 specific pathogen-free (SPF) male Sprague Dawley (SD) rats, 10–12 weeks, weighing 300–350 g, and randomly divided into the control and depression groups, with 6 animals in each group. The animals were provided by the Experimental Animal Center of the Chinese Academy of Military Medical Sciences, kept in the experimental animal department of Tianjin Medical University with 12 h of light (started at 7:00 am), 12 h of darkness, a room temperature of 25 ± 2°C and humidity ranging from 50 to 55%. Rats were divided into groups with 4 rats per cage. Food and water were available *ad libitum*. Based on the experimental methods of Willner (1998), the CUMS procedure was conducted on the depression group for 21 days. Six kinds of stressors, including ice water swimming (4°C, 5 min), water and food deprivation (24 h), tail pinch (1 min), reversed light/dark cycle (24 h), cage tilt (12 h), and wet padding (12 h), were applied in random order, with each stressor performed once a week. The control group did not receive any intervention.

We examined CUMS depression in rats by the sucrose preference test (SPT) and forced-swim test (FST). Compared with the control group, the sucrose preference rate of the depression group decreased, and the FST immobility time increased significantly. These results suggested that CUMS-depressed rats were successfully prepared.

### Surgery

The surgical procedures were performed according to Bai et al. (2012). The rats were deeply anesthetized with pentobarbital sodium (40 mg/kg, i. p.). The coordinates were determined according to the rat brain atlas in stereotaxic coordinates (Paxinos and Watson, 2007). Then two 16-channel microelectrode arrays (arranged in 2 × 8 configuration, 50 μm diameter nickel-chromium wires with formvar insulation (California Fine Wire Co., Grover Beach, CA, United States), 200 μm inter-electrode spacing, impedance <1 MΩ tested in Omega-Tip-Z (World Precision Instruments, Sarasota, FL, United States) were, respectively, implanted into mPFC (2.5–4.5 mm anterior to bregma, 0.2–1.0 mm lateral to midline, 2.5–3.0 mm from dura) and BLA (1.56–3.36 mm posterior to bregma, 4.4–5.2 mm lateral to midline, 8.6–9.1 mm from dura) (Supplementary Figure 1) of rat. Iodophor was used to prevent infection after surgery. Rats were allowed to recover from surgery for 1 week, then the electrophysiological signals were recorded from mPFC and BLA during OFT.

### Exploratory Behavior in Open Field

**FIGURE 1:**
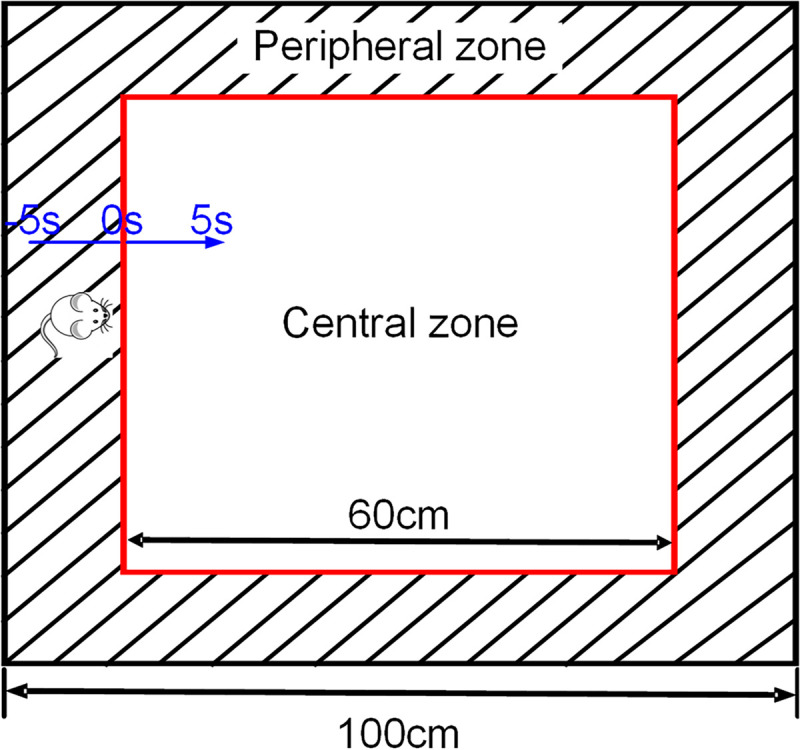
Schematic representation of exploratory behavior in OFT. The black square refers to the four walls of the open field, and the red square in the middle is the central area (only for video analysis). The area between the two squares is the peripheral zone. The blue one-way arrow indicates that the rat moves from the peripheral to the central zone, and the black two-way arrow is used to describe the size of the open field.

The open field test (OFT) is based on the rodent locomotion that drove by exploratory (curiosity) and they have a tendency to spend the majority of their time in close proximity to the walls (Hall and Ballachey, 1932; Hall, 1934). As shown in Figure 1, the open field we used in this research was square (100 cm × 100 cm), a square area (60 cm × 60 cm) in the center of the open field was defined as “central zone,” the area between the wall and “central zone” was defined as “peripheral zone.” Testing was recorded by a video tracking system (Sony, Japan) for 5 min under normal illumination. The moment when the four paws of the rat entering the central zone is defined as the reference point (RP, “0 s”), and the entry of the rats from the peripheral zone into the central zone during OFT was defined as a trial of exploratory behavior (including 5 s in the peripheral zone and 5 s in the central zone). EthoVision XT 8.5 software (Noldus, Netherlands) was used to detect the position of the rat in the open field. After the test, the open field was thoroughly cleaned with 75% alcohol to eliminate odors. Behavioral parameters, including the movement distance, the rearing times, the entries of the central zone and the time spent in the central zone, were measured.

### Neurophysiological Recording

The electrophysiological signals were collected by a neurophysiological recording system (Plexon, United States), and the rat’s position was simultaneously recorded by the overhead camera. The LFPs (16 channels each in the mPFC and BLA) were amplified (gain: 5000), bandpass filtered (0.3–120 Hz) and acquired at a frequency of 2 kHz (Tiaotiao et al., 2014). A 50-Hz notch filter was applied to eliminate noise from the main power line and correct the baseline drift by polynomial fitting. Then, the delta (1–4 Hz), theta (4–12 Hz), beta (12–30 Hz), low-gamma (30–70 Hz) and high-gamma (70–120 Hz) bands were obtained by bandpass filtering. To ensure the availability of data, the recorded data that the rat stayed in the central zone for less than 5 s were excluded from analysis.

### Information Transmission Analysis

The IF was estimated according to the directed transform function (DTF) to measure information transmission. The DTF was defined in the framework of multiple auto-regressive (MVAR) models and was used to analyze causality in the frequency domain (Seth, 2010). Based on the multivariate Granger causality connectivity analysis, the nodes of the causal network were defined as electrodes, and the edges of the causal network were elements of the DTF matrix, which were used to describe the network connectivity of the LFP. The DTF value was estimated in a window with a width of 1000 ms and an interval of 250 ms throughout the task.

According to the MVAR model, a multichannel LFPs can be represented as a data vector *X* of *N* source signals:

(1) $$X\,\left(t\right)={\left[x_{1}\,\left(t\right),x_{2}\,\left(t\right),\ldots ,x_{N}\,\left(t\right)\right]}^{T}$$

The MVAR model can then be expressed as:

(2) $$X\,\left(t\right)=\sum_{n=1}^{p}A_{n}\,X\,\left(t-n\right)+E\,\left(t\right)$$

Where *p* is the model order, calculated by the Bayesian information criterion. *A*_*n*_ is the coefficient matrix of the MVAR model. *E*(*t*) is the vector of multivariate zero mean uncorrelated white noise at time *t*.

(3) $$X\,\left(f\right)=A^{-1}\,\left(f\right)\,E\,\left(f\right)=H\,\left(f\right)\,E\,\left(f\right)$$

(4) $$H\,\left(f\right)=A^{-1}\,\left(f\right)=\left(\sum_{i=0}^{p}A\,\left(i\right)\,e^{-j\,2\,\pi \,f\,i\,\Delta \,t}\right)$$

*f* denotes a specific frequency, *H*(*f*) is the transfer function matrix, and *A*(0) = −*I*, *I* is an identity matrix.

(5) $$\gamma_{i\,j}\,\left(f\right)=\frac{{\left|H\,\left(f\right)\right|}^{2}}{\sum_{m=1}^{k}{\left|H_{i\,m}\,\left(f\right)\right|}^{2}}$$

γ_*ij*_(*f*) represents the ratio between the effect from channel *j* to channel *i* and the combined effect from all other nodes to node *j*, *k* is the number of nodes, and *H* is the transfer matrix of the system.

(6) $$D\,T\,F=\frac{1}{N\,\left(N-1\right)}\,\sum_{i\neq j\in G}D\,T\,F_{i\,j}$$

*DTF*_*ij*_ refers to the connection strength from channel *i* to channel *j*. *N* is the number of channels. *G* is the set of channels in the causal network.

The mPFC-BLA causal network was constructed based on the connection strength matrix, and the bidirectional information flow (IF) from mPFC to BLA and BLA to mPFC was calculated, respectively:

(7) $$I\,F_{P\to B}=\frac{1}{N_{P}\times N_{B}}\,\sum_{i\in N_{B}}\sum_{j\in N_{P}}D\,T\,F_{i\,j}$$

(8) $$I\,F_{B\to P}=\frac{1}{N_{B}\times N_{P}}\,\sum_{i\in N_{P}}\sum_{j\in N_{B}}D\,T\,F_{j\,i}$$

*IF*_*B→P*_ refers to the IF from the mPFC to the BLA, and *IF*_*B→P*_ indicates the IF from the BLA to the mPFC. *N*_*P*_ and *N*_*B*_ are the number of channels in the mPFC and BLA, respectively (Xia et al., 2019).

### Histology

At the completion of all behavioral and electrophysiological studies, the rats were deeply anesthetized, then transcardially fused with phosphate-buffered saline (PBS) and 4% paraformaldehyde solution. The brain was dissected and cryoprotected (30% sucrose in PBS, 4°C). Brain slices (150 μm thick) were obtained on a vibratome (Vibratome, United States), and tracks caused by electrode tips were observed on an optical microscope (Olympus, Japan). The recorded sites were verified histologically by referring to the rat brain atlas (Paxinos and Watson, 2007).

### Statistical Analysis

We collected a total of 240 trials (120 trials with the control group and 120 trials with the depression group; the distribution of trials in control and depression groups for per subject were shown in Supplementary Table 1). SPSS 22.0 was used for statistical analysis. The data in the text and figures are expressed as the mean ± SEM. The data involved in this study were all normally distributed, and *t*-test (paired sample *t*-test/independent sample *t*-test) was used to assess significant differences. Specifically, comparisons of the behavioral parameters of the two groups of rats in the OFT were performed using independent sample *t*-tests. The average theta band DTF/IF in the peripheral and central zone were compared by using paired sample *t*-test. The average theta band DTF/IF in the control and depression groups were compared by using independent sample *t*-test. *p*-values are marked statistically significant as follows: ns *p* > 0.05 no significance, ^∗^*p* < 0.05, ^∗∗^*p* < 0.01, ^∗∗∗^*p* < 0.001.

## Results

### Behavioral Performance in OFT

**FIGURE 2:**
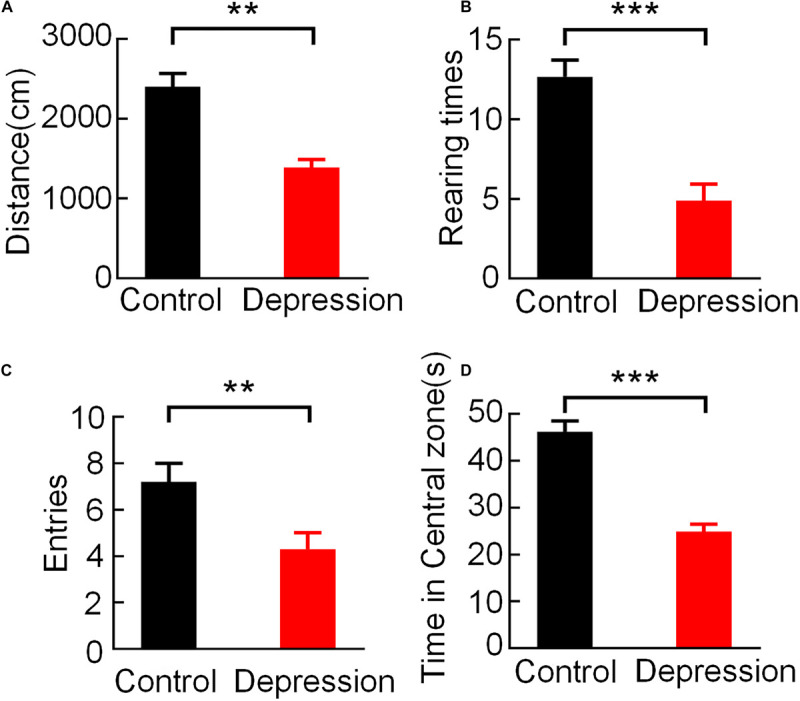
Comparison of behavioral performance in the OFT between the control and depression groups. **(A)** Comparison of the averaged movement distance. **(B)** Comparison of the averaged rearing times. **(C)** Comparison of the averaged entries of the central zone. **(D)** Comparison of the averaged time spent in the central zone. Error bars indicate SEM. Independent sample *t*-test, **p < 0.01, ***p < 0.001.

During the 5-min open field test, the behavioral parameters in the two groups of rats showed significant differences, and the average movement distance (*t* = 4.423, *p* < 0.01; Figure 2A), rearing times (*t* = 7.400, *p* < 0.001, Figure 2B), entries of the central zone (*t* = 4.676, *p* < 0.01; Figure 2C) and time spent in the central zone (*t* = 5.742, *p* < 0.001; Figure 2D) of the depression group were significantly lower than those of the control group.

### Information Transmission Between the mPFC and BLA

#### Connection Strength of the mPFC and BLA LFP Network

**FIGURE 3:**
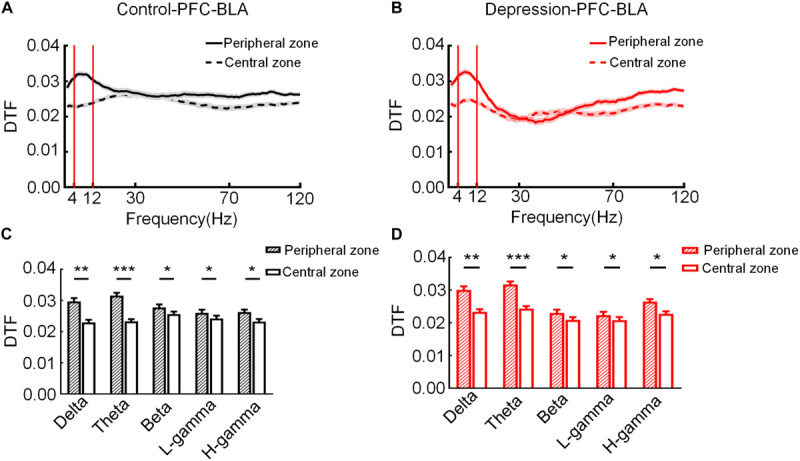
Direct transfer function (DTF) at different frequencies of the mPFC-BLA network in the peripheral and central zone. **(A)** DTF in the peripheral and central zone of the control group. **(B)** DTF in the peripheral and central zone of the depression group. The bandwidth calculated for DTF ranged from 1 to 120 Hz. **(C)** DTF of the control group in different frequency bands in the peripheral and central zone. **(D)** DTF of the depression group in different frequency bands in the peripheral and central zone. Error bars indicate SEM. Paired sample *t*-test, *p < 0.05, **p < 0.01, ***p < 0.001.

To compare mPFC and BLA information transmission between the control group and the depression group during exploratory behavior, the connection strength of mPFC and BLA LFP networks in two groups were calculated. We recorded mPFC and BLA (16 channels each) LFPs in the two groups during the OFT. LFPs from the mPFC and BLA were used to calculate and obtain the DTF of different frequencies of the mPFC-BLA network in the peripheral and central zone (Figures 3A,B). The results showed that the theta band (4–12 Hz) DTF of the mPFC and BLA changed most sharply in the peripheral and central zone (Control: Delta: *t* = 2.953, *p* < 0.01; Theta: *t* = 6.341, *p* < 0.001; Beta: *t* = 3.662, *p* < 0.05; Low-gamma: *t* = 3.369, *p* < 0.05; High-gamma: *t* = 3.522, *p* < 0.05; Figure 3C) (Depression: Delta: *t* = 3.126, *p* < 0.01; Theta: *t* = 7.255, *p* < 0.001; Beta: *t* = 2.912, *p* < 0.05; Low-gamma: *t* = 3.431, *p* < 0.05; High-gamma: *t* = 3.563, *p* < 0.05; Figure 3D) in the open field, and the theta band in the mPFC and BLA was determined to be the characteristic band of exploratory behavior.

**FIGURE 4:**
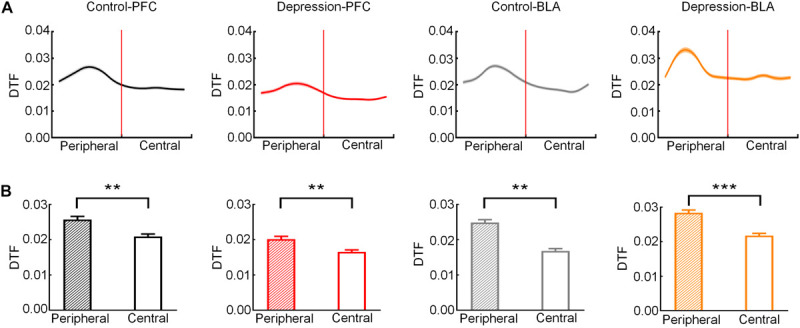
Direct transfer function (DTF) in the mPFC and BLA of the control and depression groups in the peripheral and central zones. **(A)** Averaged DTF in the theta band from the peripheral to the central zone. The red line represents the RP. **(B)** Comparison of averaged theta band DTF in peripheral and central zone. Error bars indicate SEM. Paired sample *t*-test, **p < 0.01, ***p < 0.001.

**FIGURE 5:**
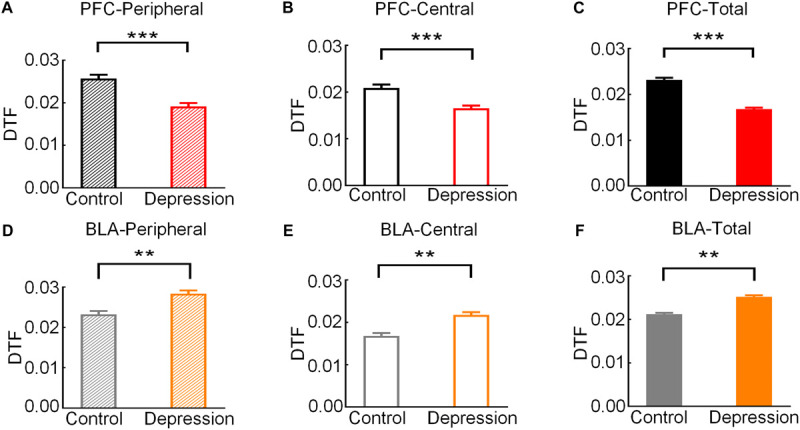
Comparison of the average theta band DTF of the mPFC and BLA in exploratory behavior between the control and depression groups. **(A)** Comparison of the average theta DTF of the mPFC in the peripheral zone. **(B)** Comparison of the average theta band DTF of the mPFC in the central zone. **(C)** Comparison of the average theta band DTF of the mPFC from the peripheral to the central zone. **(D)** Comparison of the average theta band DTF of the BLA in the peripheral zone. **(E)** Comparison of the average theta band DTF of the BLA in the central zone. **(F)** Comparison of the average theta band DTF of the BLA from the peripheral to the central zone. Error bars indicate SEM. Independent sample *t*-test, **p < 0.01, ***p < 0.001.

Then, we analyzed the theta band connection strength of the mPFC and BLA during exploratory behavior. As shown in Figure 4A, DTF in the theta band declined before rats left the peripheral zone. The theta band DTF in the peripheral zone was significantly higher than that in the central zone (Control-PFC: *t* = 3.083, *p* < 0.01; Depression-PFC: *t* = 3.235, *p* < 0.01; Control-BLA: *t* = 3.151, *p* < 0.01; Depression-BLA: *t* = 5.745, *p* < 0.001; Figure 4B), suggesting that the theta band connection strength of mPFC and BLA was stronger in the peripheral zone than in the central zone. The average theta DTF of mPFC in depression group decreased significantly compared with the control group (Peripheral zone: *t* = 6.446, *p* < 0.001; Central zone: *t* = 6.628, *p* < 0.001; Total: *t* = 6.401, *p* < 0.001; Figures 5A–C), but the average theta DTF of BLA in the depression group increased significantly in both the peripheral and central zones (Peripheral zone: *t* = 4.569, *p* < 0.01; Central zone: *t* = 4.073, *p* < 0.01; Total: *t* = 4.358, *p* < 0.01; Figures 5D–F). This indicates that the difference in the theta band connection strength of the mPFC and BLA between the control group and the depression group may be related to the abnormal information transmission between the mPFC and BLA.

#### Abnormal mPFC-BLA Information Transmission in Depression

**FIGURE 6:**
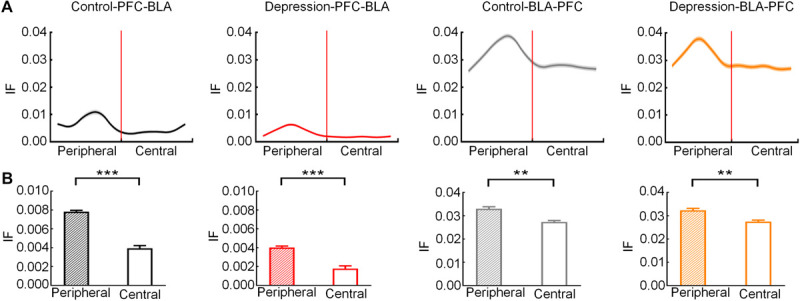
Information flow (IF) of the mPFC-BLA and BLA-mPFC between the control and depression groups in the peripheral and central zones. **(A)** Averaged IF in the theta band from the peripheral zone to the central zone. The red line represents the RP. **(B)** Comparison of averaged theta IF in the peripheral and central zone. Error bars indicate SEM. Paired sample *t*-test, **p < 0.01, ***p < 0.001.

**FIGURE 7:**
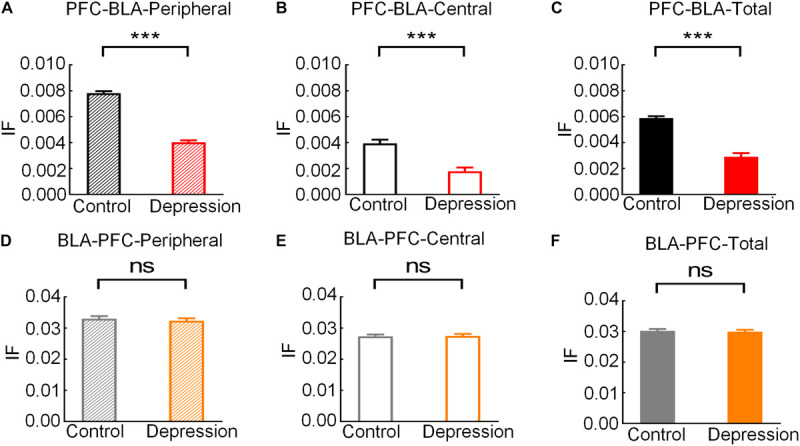
Comparison of the average theta IF of the mPFC-BLA and BLA-mPFC in exploratory behaviors between the control and depression groups. **(A)** Comparison of the average theta IF of mPFC-BLA in the peripheral zone. **(B)** Comparison of the average theta IF of the mPFC-BLA in the central zone. **(C)** Comparison of the average theta IF of the mPFC-BLA from the peripheral to the central zone. **(D)** Comparison of the average theta IF of the BLA-mPFC in the peripheral zone. **(E)** Comparison of the average theta IF of the BLA-mPFC in the central zone. **(F)** Comparison of the average theta IF of the BLA-mPFC from the peripheral to the central zone. Error bars indicate SEM. Independent sample *t*-test, ns *p* > 0.05 no significance, ***p < 0.001.

The IF was estimated based on the DTF to measure mPFC and BLA information transmission. The bidirectional IF in the theta band of the mPFC and BLA rose to a peak in the peripheral zone and then gradually decreased (Figure 6A). The average IF of the mPFC-BLA and BLA-mPFC in the theta band in the peripheral zone was significantly higher than that in the central zone (Control-PFC-BLA: *t* = 6.271; *p* < 0.001; Depression-PFC-BLA: *t* = 5.991, *p* < 0.001; Control-BLA-PFC: *t* = 4.754, *p* < 0.01; Depression-BLA-PFC: *t* = 4.498, *p* < 0.01; Figure 6B). The average theta IF of the mPFC-BLA in the depression group was significantly lower than that in the control group (Peripheral zone: *t* = 5.011, *p* < 0.001; Central zone: *t* = 4.545, *p* < 0.001; Total: *t* = 4.762, *p* < 0.001; Figures 7A–C), while the average theta IF of the BLA-mPFC in the two groups was not significantly different (Peripheral zone: *t* = 0.759, *p* > 0.05; Central zone: *t* = 0.333, *p* > 0.05; Total: *t* = 0.589, *p* > 0.05; Figures 7D–F). This indicates that mPFC-BLA information transmission is necessary for exploratory behavior, and the reduction of mPFC-BLA information transmission may inhibit exploratory behavior in depressed rats. The BLA-mPFC is not necessary for the exploratory behavior of rats.

### Histology

**FIGURE 8:**
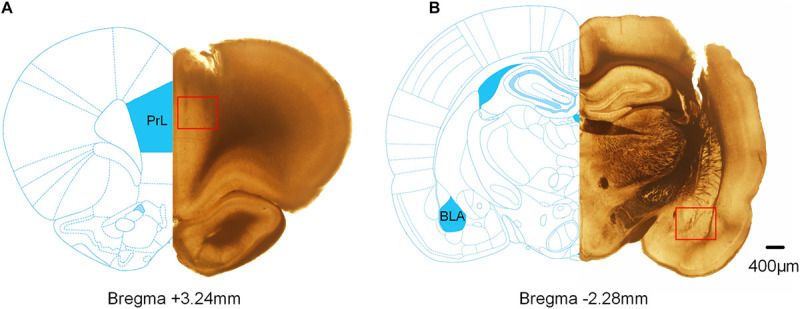
Histological verification of the recording sites in the mPFC and BLA. The superimposed schematics show the coronal brain sections at 3.24 mm anterior and 2.28 mm posterior to the bregma. The red square refers to the track of the electrode tips. **(A)** The track of the recording electrode in the mPFC. **(B)** The track of the recording electrode in BLA.

Extracellular electrophysiological recordings were performed from the mPFC and BLA of rats. Figure 8 shows representative histological sections of the recording sites (the scar caused by the electrode tip) of the mPFC and BLA.

### Correlation Between DTF/IF and Movement Speed

**FIGURE 9:**
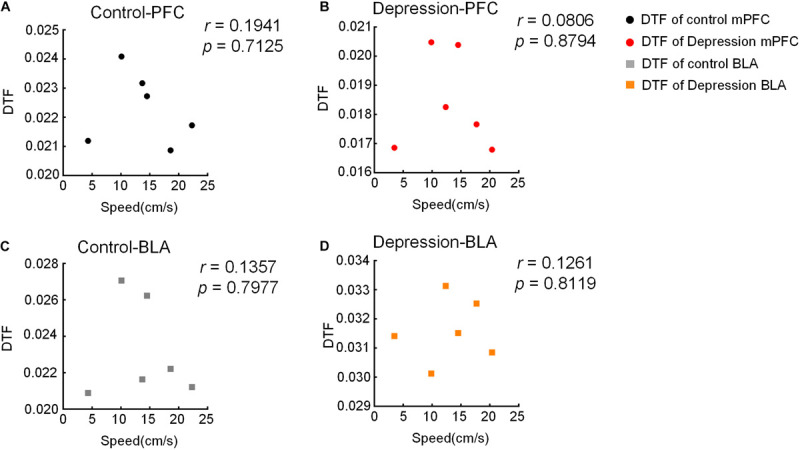
Correlation between moving speed and DTF in the OFT. **(A,B)** There is no significant correlation between moving speed and DTF of the mPFC in the **(A)** control and **(B)** depression group. **(C,D)** There is no significant correlation between moving speed and DTF of the BLA in the **(C)** control and **(D)** depression group.

**FIGURE 10:**
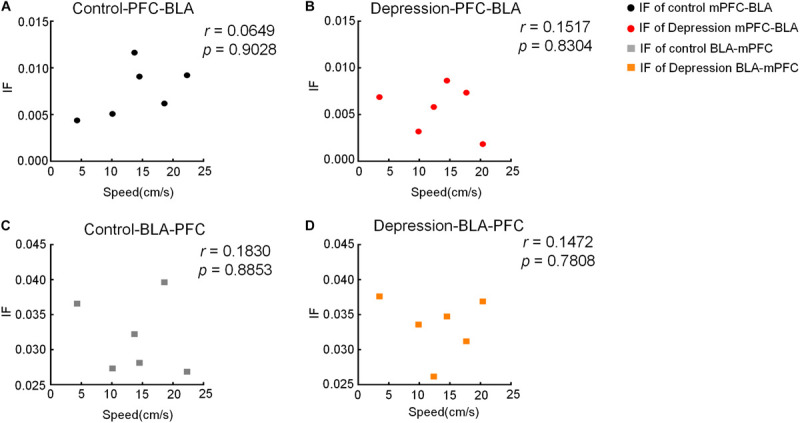
Correlation between moving speed and IF in the OFT. **(A,B)** There is no significant correlation between moving speed and IF of the mPFC-BLA in the **(A)** control and **(B)** depression group. **(C,D)** There is no significant correlation between moving speed and IF of the BLA-mPFC in the **(C)** control and **(D)** depression group.

To detect whether the movement speed of rats affects DTF/IF, the Pearson Correlation Coefficient (*r*) was calculated between the movement speed and each animal’s DTF/IF, and the results showed that the dynamic changes in DTF/IF had no significance with the movement speed of rats (Figures 9, 10).

## Discussion

Our main finding in this study was that the reduction of the theta band information transmission of mPFC-BLA in depressed rats caused a decline in mPFC network connection strength and a rise in BLA network connection strength compared to normal rats, which led to disorders of exploring behavior in depressive rats. It also shows that chronic stress not only leads to depression-like behavior but also inhibits exploratory behavior. Our results were based on the OFT behavioral parameters in two groups of rats, consistent with previous reports (Zhan, 2015; Song et al., 2018). These results quantitatively reveal the mPFC and BLA information transmission and may provide a new perspective for the study of depression.

In most individuals, the circuit of the mPFC-BLA may be specially protected from the harmful effects of stress, but when there is chronic stress, the pathway is easily affected by stress. The neuronal structure of the entire mPFC can be altered by stress, including the anterior cingulate gyrus, lower limbs and forelimb regions (Radley et al., 2004; Izquierdo et al., 2006; Shansky and Morrison, 2009). BLA projection neurons selectively avoid stress-induced remodeling, and this circuit may be prepared for increased input and possible excessive stimulation, which may lead to dysfunction (Dilgen et al., 2013). The mPFC-BLA circuit is essential for depression-like and exploratory behavior. The activation of this circuit in mice by optogenetic technology can reduce the immobility time of the FST and improve exploratory behavior in the OFT but has no effect on the sucrose preference of the SPT (Hare et al., 2019). According to the connection type between the BLA and mPFC, the BLA projection neurons are divided into two clusters: one cluster is interconnected with the mPFC (mPFC ↔ BLA), and the other only receives mPFC input (mPFC → BLA) (Zhang et al., 2019). However, chronic stress selectively shifts the mPFC-driven excitatory-inhibitory balance toward excitement in the mPFC → BLA, rather than excitement in the mPFC ↔ BLA, which is due to presynaptic glutamate release selectively increasing the results of the mPFC → BLA. Our results quantify this conclusion and explore the influence of chronic stress on mPFC-PrL and BLA during exploratory behaviors.

BLA projects glutamate into multiple subregions of the mPFC, including the anterior cingulate cortex, anterior marginal cortex, and marginal subcortex. *In vivo* electrophysiological analysis of this pathway shows that BLA activation can regulate the activity of different subpopulations of mPFC neurons (Pérez-Jaranay and Vives, 1991; Ishikawa and Nakamura, 2003). This circuit is also related to many cognitive and emotional processes, such as the acquisition and elimination of organized fear (Maren and Quirk, 2004). The circuit of the BLA-mPFC is considered to be the basis of emotional and cognitive disorders in diseases such as schizophrenia, depression and drug addiction (Shenton et al., 2001; Drevets, 2003; Bechara, 2005). The BLA-mPFC is also involved in the regulation of anxiety-like behavior and social interaction. Activating this circuit will lead to increased anxiety and social disorders in rodents, and inhibition of this circuit will produce anti-anxiety effects and promote social interaction (Felix-Ortiz et al., 2016).

In addition to the mPFC and BLA, the hippocampus (HPC) is also considered to be a key brain region for depression and exploratory behavior (Voss et al., 2011; Lu et al., 2019). The circuit of the HPC-mPFC originates from the ventral hippocampus (vHPC) CA1 and the lower support, projecting to PrL and MO of the mPFC (Thierry et al., 2000). Research has shown that activation of HPC-PFC can produce antidepressant effects (Carreno et al., 2016). Due to the development of experimental technology, more brain regions and circuits have become potential targets for studying emotion (Camporeze et al., 2018). The dorsal nucleus raphe, globus pallidus and various circuits and networks composed of the above brain regions are considered potential research directions for regulating emotions and cognition (Chang and Grace, 2014).

Taken together, our results indicate that mPFC-BLA information transmission is necessary for exploratory behavior and provide new ideas and potential mechanisms for depression research. Future research will involve more brain regions, more signal processing algorithms, and other behavioral tasks.

## Data Availability Statement

The datasets presented in this article are not readily available because the raw/generated data required to reproduce these findings cannot be shared at this time as the data also forms part of an ongoing study. Requests to access the datasets should be directed to XZ.

## Ethics Statement

The animal study was reviewed and approved by the Tianjin Medical University Animal Care and Use Committee.

## Author Contributions

XZ and TL designed the experiments. CQ, WB, and ZW carried out the experiments and analyzed the data. CQ and XZ wrote the manuscript. All authors read and approved the final manuscript.

## Conflict of Interest

The authors declare that the research was conducted in the absence of any commercial or financial relationships that could be construed as a potential conflict of interest.
