# Supplementary material for: Reduced Information Transmission of Medial Prefrontal Cortex to Basolateral Amygdala Inhibits Exploratory Behavior in Depressed Rats
Source: Front Neurosci. 2020 Dec 3;14:608587. doi: 10.3389/fnins.2020.608587 (PMC7744617; doi:10.3389/fnins.2020.608587)
Supplement: Supplementary file 1 [file Table_1.DOCX]

## Supplementary Table

**Supplementary Table 1**

Distribution of trials in control and depression groups for per subject.

| Control group | |  | Depression group | |
| --- | --- | --- | --- | --- |
| Rat | Trials |  | Rat | Trials |
| 1 | 22 |  | 1 | 20 |
| 2 | 20 |  | 2 | 18 |
| 3 | 21 |  | 3 | 19 |
| 4 | 17 |  | 4 | 22 |
| 5 | 19 |  | 5 | 21 |
| 6 | 21 |  | 6 | 20 |
| Sum | 120 |  | Sum | 120 |
