# Supplementary figures and images for: Reduced Information Transmission of Medial Prefrontal Cortex to Basolateral Amygdala Inhibits Exploratory Behavior in Depressed Rats
Source: Front Neurosci. 2020 Dec 3;14:608587. doi: 10.3389/fnins.2020.608587 (PMC7744617; doi:10.3389/fnins.2020.608587)

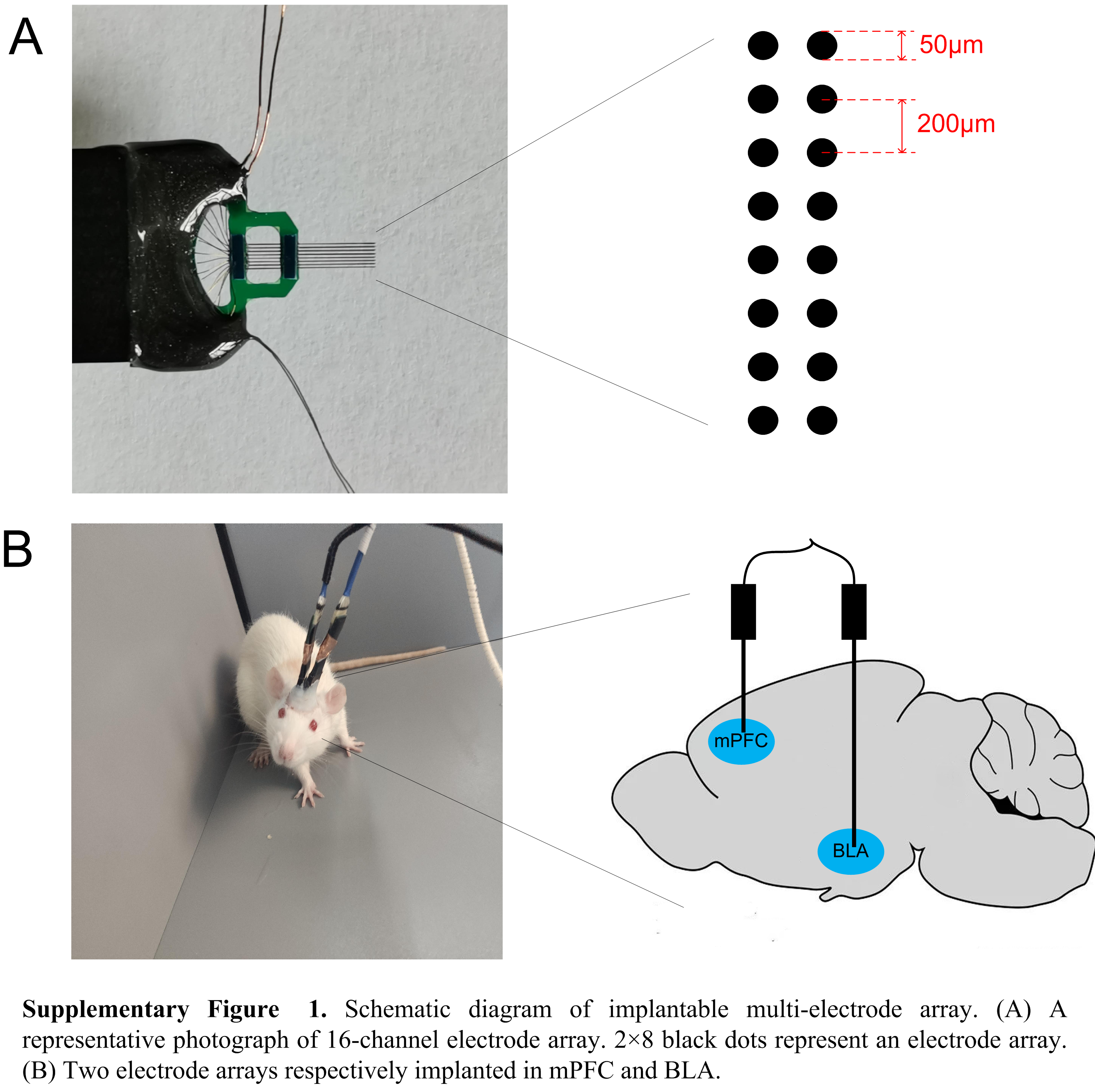

Supplement: Supplementary file 2 [file Image_1.tif]
